# Using the hydrogen and oxygen in water directly for hydrogenation reactions and glucose oxidation by photocatalysis†
†Electronic supplementary information (ESI) available. See DOI: 10.1039/c5sc03178h


**DOI:** 10.1039/c5sc03178h

**Published:** 2015-10-09

**Authors:** Baowen Zhou, Jinliang Song, Huacong Zhou, Tianbin Wu, Buxing Han

**Affiliations:** a Beijing National Laboratory for Molecular Sciences , CAS Key Laboratory of Colloid and Interface and Thermodynamics , Institute of Chemistry , Chinese Academy of Sciences , Beijing 100190 , China . Email: songjl@iccas.ac.cn ; Email: hanbx@iccas.ac.cn

## Abstract

Direct utilization of the abundant hydrogen and oxygen in water for organic reactions is very attractive and challenging in chemistry. Herein, we report the first work on the utilization of the hydrogen in water for the hydrogenation of various organic compounds to form valuable chemicals and the oxygen for the oxidation of glucose, simultaneously by photocatalysis. It was discovered that various unsaturated compounds could be efficiently hydrogenated with high conversion and selectivity by the hydrogen from water splitting and glucose reforming over Pd/TiO_2_ under UV irradiation (350 nm). At the same time, glucose was oxidated by the hydroxyl radicals from water splitting and the holes caused by UV irradiation to form biomass-derived chemicals, such as arabinose, erythrose, formic acid, and hydroxyacetic acid. Thus, the hydrogen and oxygen were used ideally. This work presents a new and sustainable strategy for hydrogenation and biomass conversion by using the hydrogen and oxygen in water.

## Abstract 2

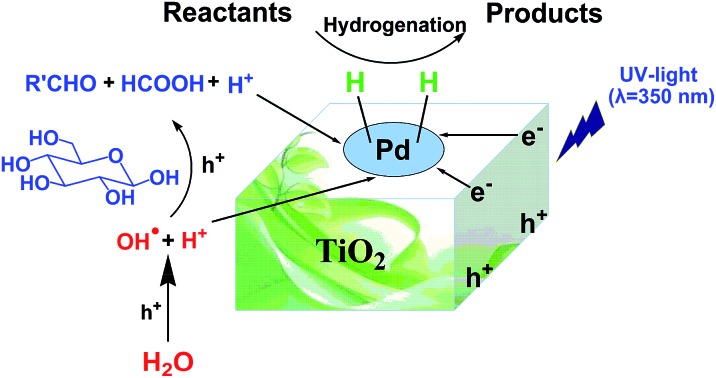
This work reports the utilization of hydrogen in water for the hydrogenation of organic compounds and the oxygen for the oxidation of glucose simultaneously by photocatalysis.

## Introduction

Efficient utilization of hydrogen and oxygen in water is one of the most attractive but challenging strategies for the sustainable development of our society. Photocatalytic water splitting offers a fascinating means for the production of H_2_ and O_2_ from water.^1^,^2^ Over the past decades, a considerable effort has been devoted to this area, and great progress has been made.^3^ However, many challenging problems are still unsolved, such as low energy efficiency, environmentally-sensitive reaction systems, separation of H_2_ and O_2_, and the efficient storage, transportation and combustion of H_2_, *etc.* Therefore, it is highly attractive to develop effective and novel routes for the utilization of hydrogen and oxygen from water splitting. Using the abundant hydrogen and oxygen in water directly for hydrogenation and oxidation is a desirable strategy.

Lignocellulosic biomass is an abundant and renewable resource.^4^ Glucose can be obtained from the depolymerization of cellulose, which is a main component of lignocellulosic biomass.^5^ Conversion of glucose into various value-added chemicals and fuels has received much attention.^6^ Furthermore, H_2_ can also be generated from glucose by various routes, including photocatalytic processes,^7^ catalytic reforming,^8^ and enzymatic procedures.^9^ Up to now, attention has been mainly focused on the transformation of glucose into chemicals or H_2_. Exploration of new processes for glucose utilization is a long term task.

Hydrogenation is an important approach for producing various chemicals. Current hydrogenation processes usually suffer from harsh reaction conditions and lower selectivity. Moreover, the H_2_ used is mainly derived from fossil resources.^10^–^12^ Furthermore, the reduction processes that use stoichiometric reductants generally form large amounts of environmentally unfriendly waste.^13^–^15^ In recent years, photocatalytic reduction has been reported to be an alternative method for the traditional hydrogenation process with high selectivity under milder reaction conditions. Triethanolamine,^16^,^17^ isopropanol,^18^ and oxalic acid,^19^*etc.*, are usually used as electron and proton donors, which makes the process less sustainable.

**Scheme 1:**
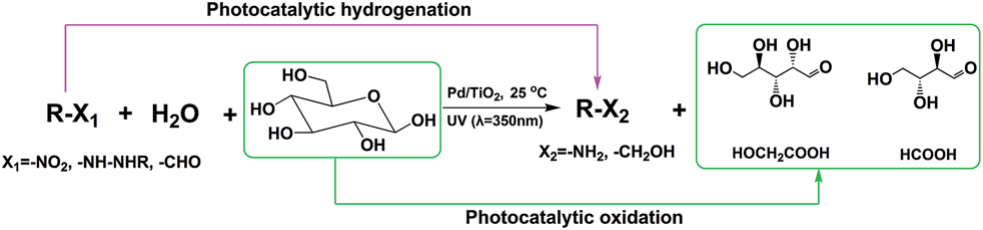
Photocatalytic water splitting for hydrogenation of various organic compounds and conversion of glucose to valuable chemicals.

The use of cheap and abundant resources in the hydrogenation of organic compounds and transformation of biomass into value-added chemicals is of great importance. Herein, we report the first work of using water to provide hydrogen and oxygen for the hydrogenation of organic compounds and oxidation of biomass into value-added chemicals simultaneously by photocatalysis. In this approach, the hydrogen in water is utilized for the hydrogenation of different organic compounds, and at the same time the oxygen in water is involved in the oxidation of glucose to generate a series of biomass-derived valuable chemicals, as shown in Scheme 1, in which the main products are provided.

## Results and discussion

**Fig. 1:**
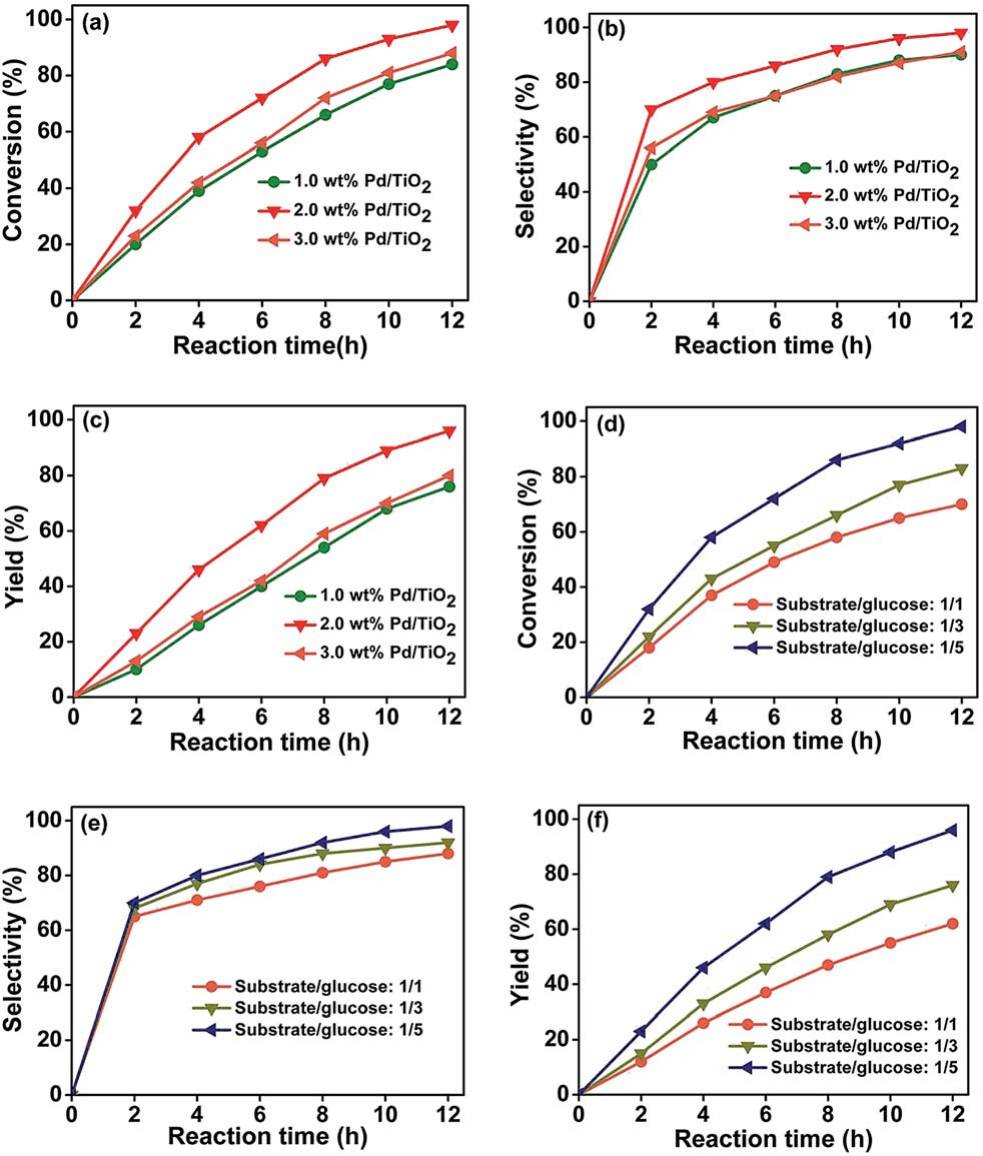
Effect of the Pd content in the Pd/TiO_2_ catalysts (a–c) and the molar ratio of glucose to substrate (d–f) on the photocatalytic reduction of 4-nitroacetophenone to 4-aminoacetophenone. Reaction conditions: 4-nitroacetophenone, 0.1 mmol; water, 1 mL; 0.025 g Pd/TiO_2_ with various Pd content; UV-light (350 nm, 4 mW cm^–2^); illuminated area, 2 cm^2^; temperature, 25 °C. The molar ratio of glucose to substrate was 5 in (a–c); Pd/TiO_2_ with 2 wt% Pd was used in (d–f).

The selective hydrogenation of nitrobenzenes containing other reducible groups provides a convenient route to produce functionalized anilines, which are high-value intermediates for various fine chemicals. Thus, the photocatalytic reduction of functionalized nitrobenzenes was selected to study the reaction in an aqueous solution of glucose by UV-light (350 nm). Pd/TiO_2_, which is effective for the photocatalytic hydrogenation of the nitro group in the presence of various alcohols,^20^,^21^ was utilized as the catalyst. Transmission electron microscope (TEM) examinations indicated that the difference between the size of the Pd nanoparticles (PdNPs) in the Pd/TiO_2_ catalysts with Pd loadings of 1 wt%, 2 wt%, and 3 wt% was not considerable (Fig. S1†). Fig. 1a–c shows the influence of the Pd content in the Pd/TiO_2_ catalysts on the photocatalytic reduction of 4-nitroacetophenone to 4-aminoacetophenone. It was found that the catalyst with 2 wt% Pd had a better catalytic performance than those with 1 wt% and 3 wt% Pd. The main reason for this is that the loading amount of Pd affected the reaction in opposite ways. In the catalytic cycle, the Pd nanoparticles served as electron sinks and provided active sites for forming H-PdNPs, which are the active species for the reduction of the nitro group. On the other hand, excessive loading of Pd nanoparticles could reduce the photocatalytic activity because Pd could also act as charge carrier recombination centers,^22^ and shield the incident light absorption by the semiconductor.^23^ The competition of the above factors resulted in the catalyst with 2 wt% Pd content having the best performance. The 4-nitroacetophenone could be completely converted in 12 hours (Fig. 1a) with a 4-aminoacetophenone selectivity of 98% (Fig. 1b).

Fig. 1d–f illustrates the influence of the molar ratio of glucose to substrate on the photocatalytic hydrogenation of 4-nitroacetophenone with different reaction times. It can be seen that the conversion of 4-nitroacetophenone (Fig. 1d), and the selectivity (Fig. 1e) and yield (Fig. 1f) of 4-aminoacetophenone increased with the increasing molar ratio of glucose to substrate. During the reaction process, glucose performed as the scavenger of photoexcited holes to inhibit the recombination of the electron–hole pairs on TiO_2_. More importantly, the photocatalytic reforming of glucose with the aid of hydroxyl radicals from water splitting could release protons, which could be reduced by photoinduced electrons to form the active H-PdNP species, this will be discussed in detail in subsequent sections. Therefore, increasing the molar ratio of glucose to substrate facilitated the elimination of the holes and promoted the formation of the H-PdNP species, which was beneficial to the photocatalytic reduction. Our control experiment showed that the hydrogenation reaction could be prohibited by the addition of 2,2′,6,6′-tetramethylpiperidine-*N*-oxyl (TEMPO, 100 mg), which could remove hydrogen atoms from the metal surfaces,^24^ indicating the formation of H-PdNPs. In addition, when the molar ratio of glucose to substrate was increased to 100/1, the reaction time for the complete conversion of 4-nitroacetophenone was reduced to 4 hours with a 4-aminoacetophenone selectivity of 98%, which further proved that a high molar ratio of glucose to substrate was beneficial for the reaction.

**Fig. 2:**
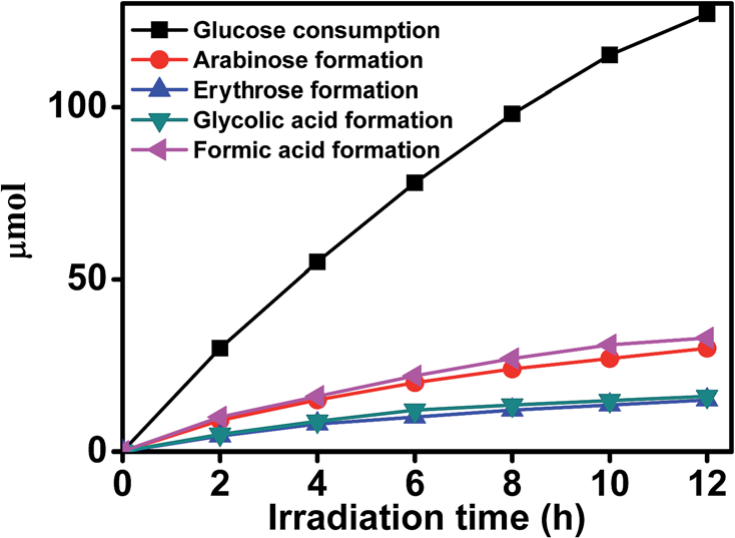
The dependence of the amounts of the products from glucose reforming in water on time with the reaction conditions of Fig. 1b.

Water splitting accompanied with glucose reforming continuously proceeded over Pd/TiO_2_ under UV-light illumination, releasing protons and electrons for photocatalytic hydrogenation. As shown in Fig. 2 and Table 1, 0.13 mmol of glucose was consumed for the total conversion of 0.1 mmol of 4-nitroacetophenone to 4-aminoacetophenone, this was found from HPLC analysis with a refractive detector (Shimadzu RID-10A). Simultaneously, a series of biomass-derived chemicals, including arabinose, erythrose, formic acid, and hydroxyacetic acid were formed from the consecutive photooxidation of glucose by oxygen in the form of hydroxyl radicals from water and the holes on TiO_2_ caused by UV irradiation. In order to confirm that the hydrogen in water took part in the reaction, we carried out a control experiment using D_2_O to replace H_2_O with a reaction time of 12 hours and the other conditions were the same as those shown in Fig. 1b. The –NO_2_ was reduced to –NH_2_, –NDH, and –ND_2_ with a –NH_2_ : –NDH : –ND_2_ ratio of 0.60 : 0.32 : 0.08, indicating that 24% of the hydrogen was from water in the reaction. This indicates that the hydrogen for the reduction was derived from both water splitting and glucose reforming. Our experiment showed that the reaction did not occur without glucose, indicating that to use the hydrogen in water for the hydrogenation reaction, the oxygen from water must be consumed in the oxidation of glucose simultaneously, this will be discussed in the following sections. In addition, hydrogenation did not occur in the dark or when the wavelength of the incident light was >420 nm. The reason for this was that the electron–hole separation resulting from the wide band gap of TiO_2_ (3.2 eV) could not be induced, which was the first key step for the subsequent photoredox reactions. Furthermore, only a very little amount (about 2%) of 4-aminoacetophenone was produced upon simulated sunlight because UV light energy is only a small proportion (about 4%) of solar energy. These results suggested that UV-light was the driving force of the reaction when Pd/TiO_2_ was used as the catalyst.

**Table 1:** Photocatalytic selective reduction of various nitro compounds in water–glucose system^*a*^

Entry	Substrate	Time (h)	Nitroarenes con.^*b*^ (%)	Anilines sel.^*c*^ (%)	Glu. cons.^*d*^ (mmol)
1	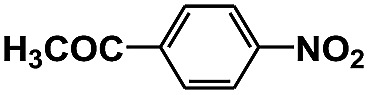	12	>99	99	0.13
2	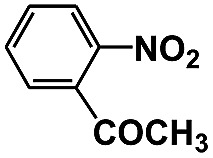	12	92	89	0.13
3	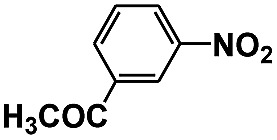	12	98	96	0.12
4	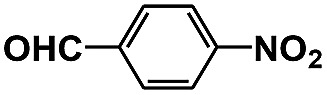	11	>99	98	0.13
5	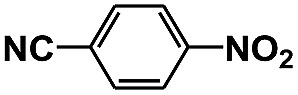	10	>99	99	0.11
6	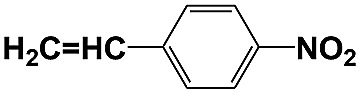	16	98	93	0.12
7	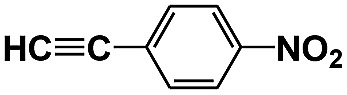	18	>99	96	0.12
8	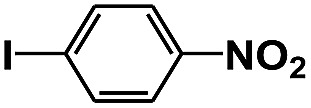	16	97	88	0.13
9	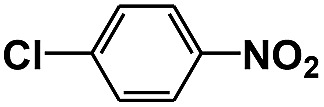	16	95	90	0.13
10	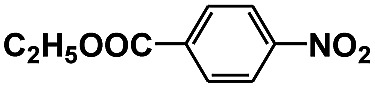	20	98	96	0.12
11	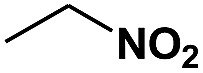	48	95	97	0.20

^*a*^Reaction conditions: substrates, 0.1 mmol; glucose, 0.5 mmol; water, 1 mL; temperature, 25 °C; 0.025 g Pd/TiO_2_ with 2 wt% Pd; UV-light (350 nm, 4 mW cm^–2^); illuminated area, 2 cm^2^.

^*b*^Con. = conversion.

^*c*^Sel. = selectivity.

^*d*^Glu. cons. = glucose consumption.

**Fig. 3:**
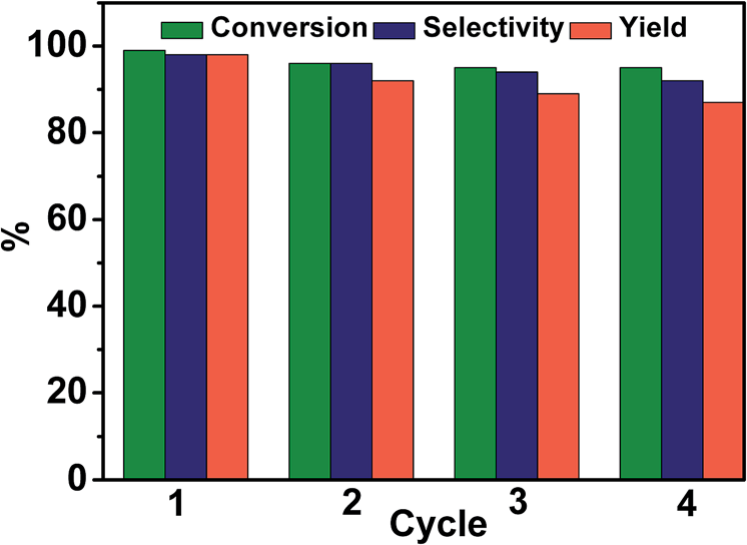
Reusability of Pd/TiO_2_ for photocatalytic reduction of 4-nitroacetophenone in glucose aqueous solution. Reaction conditions: 4-nitroacetophenone, 0.1 mmol; glucose, 0.5 mmol; water, 1 mL; temperature, 25 °C; reaction time, 12 h; 0.025 g Pd/TiO_2_ with 2 wt% Pd; UV-light irradiation (350 nm, 4 mW cm^–2^); illuminated area, 2 cm^2^.

Experiments were also conducted to examine the reusability of Pd/TiO_2_. In each cycle, Pd/TiO_2_ was recovered by centrifugation and washed with ethanol. After being dried at 60 °C under vacuum for 12 hours, the catalyst was reused for the next run. The conversion of 4-nitroacetophenone and the yield and selectivity of 4-aminoacetophenone are shown in Fig. 3. It was found that there was no considerable decrease in the conversion, selectivity, and yield after four cycles, indicating that Pd/TiO_2_ was stable in the reaction. Meanwhile, Pd/TiO_2_ recovered after being reused four times was characterized by TEM, and the results indicated that the size of the Pd particles was not changed obviously (Fig. S4†).

The photocatalytic reduction of nitrobenzenes with various substituted reducible groups was performed, and the results are presented in Table 1. It was found that all the nitrobenzenes examined could be converted to the corresponding anilines in the water–glucose system. The activity order of *para*-, *meta*- and *ortho*-substituted nitroacetophenones (entries 1–3, Table 1) was *para*- > *meta*- > *ortho*-, caused mainly because of steric hindrance. Meanwhile, 4-iodo-nitrobenzene (entry 8, Table 1) and 4-chloro-nitrobenzene (entry 9, Table 1) showed lower selectivity because of a dehalogenation reaction that occurred in the reaction process. In addition, nitroethane (entry 11, Table 1) could be reduced to ethylamine with a selectivity of 97% and a prolonged reaction time (48 h), indicating that our reaction system could also be used to reduce nitroalkanes.

From Table 1, it was found that the substituted nitrobenzenes with other reducible groups could be reduced to the corresponding anilines with good to excellent (88–99%) selectivity by the economic consumption of glucose. The high selectivity can be attributed mainly to two reasons. The first is that the nitro groups prefer to be adsorbed onto the catalysts in comparison with other reducible groups,^10^ which was beneficial to the selective reduction of the nitro group. A kinetics experiment was conducted to prove this. When a mixture of nitrobenzene (0.1 mmol) and benzaldehyde (0.1 mmol) was used as the reactants, it was found that nitrobenzene was preferentially hydrogenated in the process, while the reduction of benzaldehyde was inhibited completely in the presence of nitrobenzene (Fig. S2†). Secondly, the nitro group has a stronger electron-withdrawing ability than other substituted groups, exhibiting better electrophilicity,^16^ which is favorable for the reduction of the nitro group by the active H-PdNP species when compared with other reducible groups. We designed and carried out control experiments to find evidence to support this argument. In the experiment, the photocatalytic hydrogenation of nitrobenzene (0.1 mmol), benzaldehyde (0.1 mmol) and acetophenone (0.1 mmol) was performed separately in 2 mol L^–1^ glucose aqueous solution with a reaction time of 6 hours under UV-light (350 nm), and the results are illustrated in Fig. S3.† It was found that the nitro group had a higher reaction rate than the aldehyde and carbonyl groups. This suggests that the nitro group had a higher intrinsic catalytic activity than the other reducible groups.

**Fig. 4:**
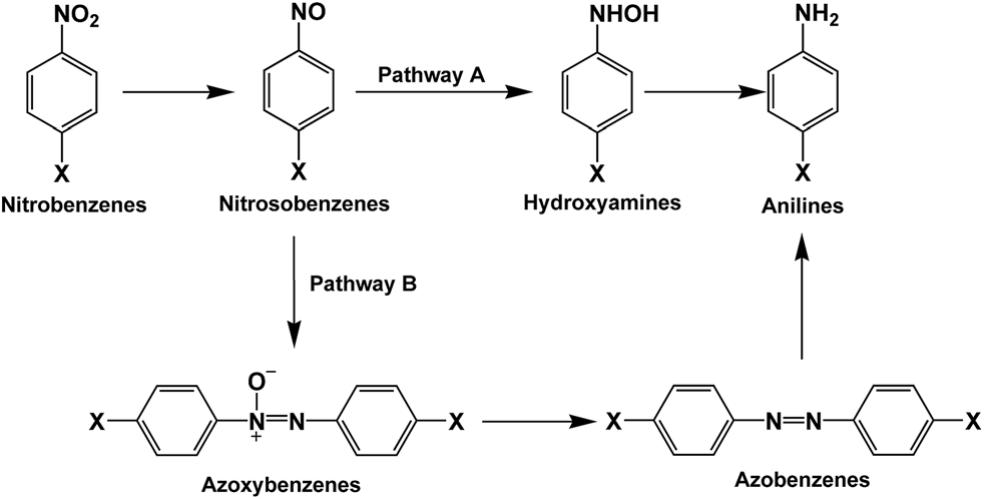
Possible pathways of photocatalytic selective reduction of functionalized nitroarenes to anilines.

**Fig. 5:**
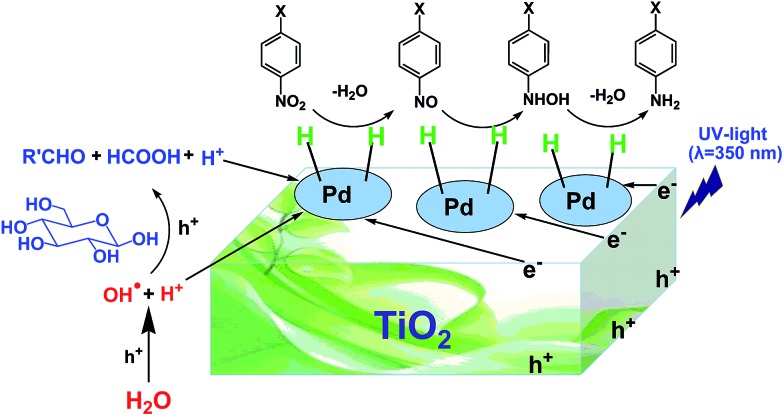
Proposed mechanism of photocatalytic water splitting and glucose reforming for selective reduction of nitrobenzenes to anilines catalyzed by Pd/TiO_2_.

**Fig. 6:**
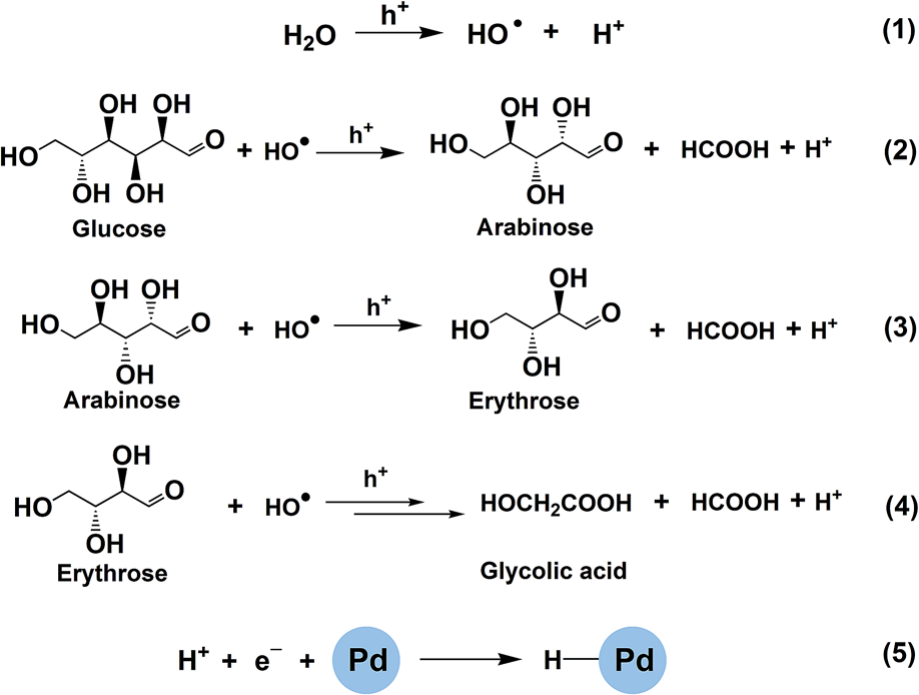
Proposed routes of photocatalytic water splitting and glucose reforming over Pd/TiO_2_.

Generally, there are two possible pathways for hydrogenating nitrobenzenes to anilines (Fig. 4). As shown by HPLC analysis, there were no azoxybenzene and azobenzene produced during the entire catalytic process. In contrast, nitrosobenzene and hydroxylamine were formed. These results indicated that the photocatalytic reduction of nitrobenzenes to the corresponding anilines was through pathway A in our reaction system (Fig. 4). On the basis of the experimental results and some related reports,^10^,^25^,^26^ we propose a possible mechanism about the photocatalytic selective reduction of nitrobenzenes to anilines in the water–glucose system over Pd/TiO_2_, which is shown in Fig. 5 and 6. Firstly, electron–hole separation on TiO_2_ occurred under UV-light irradiation (Fig. 5). Then, water splitting accompanied with the photocatalytic reforming of glucose was induced by the photogenerated holes to release protons and hydroxyl radicals (Fig. 5). The hydroxyl radicals were involved in the photocatalytic reforming of glucose. A series of biomass-derived chemicals were formed through C1–C2 cleavage from the continuous photooxidation of glucose by the hydroxyl radicals from water splitting and the holes on TiO_2_ caused by UV irradiation (Fig. 6). Subsequently, the photoinduced electrons could readily reduce the H^+^ from water splitting and glucose reforming on the PdNPs to form the active H-PdNP species (Fig. 5). Finally, the nitro group of the nitrobenzenes is preferably adsorbed onto the catalyst, and is predominantly reduced to the functionalized anilines by the H-PdNP species through pathway A shown in Fig. 4.

Inspired by the results obtained from the selective photocatalytic reduction of nitrobenzenes, we explored the hydrogenation of other kinds of unsaturated organic compounds using the same method, and the results are illustrated in Table 2. It was found that the hydrogen from water splitting and glucose reforming could be used for efficiently hydrogenating benzaldehdye and hydroazobenzene to the corresponding saturated products with high yields. The products from glucose were also mainly arabinose, erythrose, formic acid, and hydroxyacetic acid. Control reactions conducted in D_2_O showed that the content of deuterohydrogen in the products was 39% and 30%, respectively. This result further proved that the hydrogen for the reductions originated from both water and glucose.

**Table 2:** Photocatalytic water splitting and glucose reforming for various hydrogenations^*a*^

Entry	Reactant	Product	*t* (h)	Con.^*b*^ (%)	Yield (%)	Hydrogen from water
1	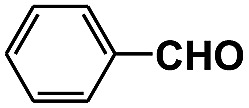	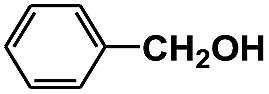	24	96	89	39%
2	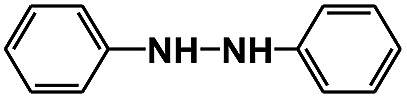	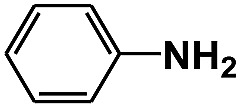	10	100	90	30%

^*a*^Reaction conditions: substrates, 0.1 mmol; glucose, 0.5 mmol; water, 1 mL; temperature, 25 °C; 0.025 g Pd/TiO_2_ with 2 wt% Pd; UV-light (350 nm, 4 mW cm^–2^); illuminated area, 2 cm^2^.

^*b*^Con. = conversion.

## Conclusions

In summary, the direct utilization of the hydrogen and oxygen in water for organic reactions was realized by photocatalysis. It was discovered that nitrobenzenes with different reducible groups could be efficiently reduced to the corresponding anilines with high selectivity by the hydrogen from water splitting and glucose reforming in the presence of Pd/TiO_2_ under UV irradiation (350 nm). Control experiments in D_2_O indicated that the hydrogen for the hydrogenation of nitrobenzenes from water was about 24%. Meanwhile, glucose was oxidated to various biomass-derived chemicals, such as arabinose, erythrose, glycolic acid, and formic acid, through C1–C2 bond cleavage by the hydroxyl radicals from water splitting and the holes on TiO_2_ caused by UV irradiation. Further study indicated that the consumption of the oxygen from water in the oxidation of glucose is necessary for the hydrogenation reactions.

## Experimental

### Preparation of Pd/TiO_2_ with different amounts of Pd

The supported catalysts were synthesized by a photoreduction method, which was similar to that reported in the literature.^27^ In a typical experiment, 5 mL of water, 0.090 g of glucose, 0.025 g of TiO_2_, and desired amounts of PdCl_2_ were added into a stainless-steel reactor of 10 mL with a top-irradiation quartz window. The Pd/TiO_2_ was obtained after the photocatalytic reduction of Pd(ii) by UV irradiation (*λ* = 350 nm, light intensity 4 mW cm^–2^) for 1 h.

### Photocatalytic reactions

The photocatalytic reaction was carried out in a cylindrical stainless-steel reactor of 10 mL. There was a quartz window at the top of the reactor for the light irradiation. In an experiment, desired amounts of water, catalyst, glucose, and substrate were added into the reactor. After degassing, the reactor was irradiated by a xenon lamp equipped with an optical filter for a desired reaction time at 25 °C, and the illuminated area was 2 cm^2^. The photoreduction products were analyzed by HPLC with a Shimadzu LC-15C pump, a Shimadzu UV-Vis SPD-15C detector, and a Hypersil ODS2 column at 35 °C. A methanol/water solution (60/40 V/V) was used as the mobile phase at a flow rate of 1.0 mL min^–1^. The chemicals in the reaction mixture were identified by GC-MS (QP-2010) as well as by comparing retention times to the respective standards in the HPLC traces.

### Reusability of Pd/TiO_2_

In the experiments to test the reusability of Pd/TiO_2_, the catalyst was recovered by centrifugation, and washed with ethanol. After drying under vacuum at 60 °C for 12 h, the catalyst was reused for the next run.

## Supplementary Material

Supplementary information

## References

[cit1] Zhao W. N., Liu Z. P. (2014). Chem. Sci..

[cit2] Zhang C. X., Chen C. H., Dong H. X., Shen J. R., Dau H., Zhao J. Q. (2015). Science.

[cit3] Hisatomi T., Kubota J., Domen K. (2014). Chem. Soc. Rev..

[cit4] Julis J., Leitner W. (2012). Angew. Chem., Int. Ed..

[cit5] He M. Y., Sun Y. H., Han B. X. (2013). Angew. Chem., Int. Ed..

[cit6] Deng L., Li J., Lai D. M., Fu Y., Guo Q. X. (2009). Angew. Chem., Int. Ed..

[cit7] Kawai T., Sakata T. (1980). Nature.

[cit8] Cortright R. D., Davda R. R., Dumesic J. A. (2002). Nature.

[cit9] Woodward J., Orr M., Cordray K., Greenbaum E. (2000). Nature.

[cit10] Boronat M., Concepcion P., Corma A., Gonzalez S., Illas F., Serna P. (2007). J. Am. Chem. Soc..

[cit11] Chen L. Y., Chen H. R., Luque R., Li Y. W. (2014). Chem. Sci..

[cit12] Westerhaus F. A., Jagadeesh R. V., Wienhofer G., Pohl M. M., Radnik J., Surkus A. E., Rabeah J., Junge K., Junge H., Nielsen M. (2013). Nat. Chem..

[cit13] Gao G., Tao Y., Jiang J. Y. (2008). Green Chem..

[cit14] Patil R. D., Sasson Y. (2015). Appl. Catal., A.

[cit15] Su Y. G., Lang J. Y., Li L. P., Guan K., Du C. F., Peng L. M., Han D., Wang X. J. (2013). J. Am. Chem. Soc..

[cit16] Yang X. J., Chen B., Zheng L. Q., Wu L. Z., Tung C. H. (2014). Green Chem..

[cit17] Sun D. R., Liu W. J., Fu Y. H., Fang Z. X., Sun F. X., Fu X. Z., Zhang Y. F., Li Z. H. (2014). Chem.–Eur. J..

[cit18] Shiraishi Y., Togawa Y., Tsukamoto D., Tanaka S., Hirai T. (2012). ACS Catal..

[cit19] Imamura K., Yoshikawa T., Nakanishi K., Hashimoto K., Kominami H. (2013). Chem. Commun..

[cit20] Selvam K., Sakamoto H., Shiraishi Y., Hirai T. (2015). New J. Chem..

[cit21] Zhou B. W., Song J. L., Zhou H. C., Wu L. Q., Wu T. B., Liu Z. M., Han B. X. (2015). RSC Adv..

[cit22] Ye M. D., Gong J. J., Lai Y. K., Lin C. J., Lin Z. Q. (2012). J. Am. Chem. Soc..

[cit23] Ran J. R., Zhang J., Yu J. G., Jaroniec M., Qiao S. Z. (2014). Chem. Soc. Rev..

[cit24] Roth J. P., Yoder J. C., Won T. J., Mayer J. M. (2001). Science.

[cit25] Chong R. F., Li J., Ma Y., Zhang B., Han H. X., Li C. (2014). J. Catal..

[cit26] Chen X. B., Shen S. H., Guo L. J., Mao S. S. (2010). Chem. Rev..

[cit27] Xie S. J., Wang Y., Zhang Q. H., Fan W. Q., Deng W. P., Wang Y. (2013). Chem. Commun..

