# Supplementary material for: Using the hydrogen and oxygen in water directly for hydrogenation reactions and glucose oxidation by photocatalysis
Source: Chem Sci. 2015 Oct 9;7(1):463–8. doi: 10.1039/c5sc03178h (PMC5952314; doi:10.1039/c5sc03178h)
Supplement: Supplementary file 1 [file SC-007-C5SC03178H-s001.pdf]

Electronic Supplementary Material (ESI) for Chemical Science.
This journal is © The Royal Society of Chemistry 2015

Supporting Information for

Using hydrogen and oxygen in water directly for hydrogenation reactions and glucose oxidation by photocatalysis

Baowen Zhou, Jinliang Song,* Huacong Zhou, Tianbin Wu and Buxing Han*

Beijing National Laboratory for Molecular Sciences, CAS Key Laboratory of Colloid and Interface and Thermodynamics, Institute of Chemistry, Chinese Academy of Sciences, Beijing 100190, China. E-mail: songjl@iccas.ac.cn, hanbx@iccas.ac.cn.

Experimental Section

Materials

Arabinose (99%), 4-nitrobenzonitrile (97%), 4-aminobenzonitrile (98%), 4-nitrobenzaldehyde (99%), ortho-aminoacetophenone (98%), ethyl-4-nitrobenzoate (98%), 1-iodo-4-nitrobenzene (98%), 4-iodoaniline (98%), 4-aminostyrene (98%), nitroethane (99%), ethylamine (70%), 4-nitroacetophenone (98%), and 4-aminoacetophenone (99%) were purchased from Alfa Aesar. $PdCl_2$ (99.5%) was obtained from Jinke Chemical Reagent Shenyang Co., Ltd, China. Hydroxyacetic acid (95%), 4-nitrophenylacetylene (97%), and 4-ethynylaniline (97%) were provided by Ark. Glucose (99%), formic acid (99%), 4-chloronitrobenzene (99%), 4-chloroaniline (99%), aniline (99%) and benzaldehyde (98%) were supplied by J&K Scientific Co., Ltd. Erythrose (75%) was provided by Aladdin. Deuterium oxide (99.9%) was offered by Beijing InnoChem Science & Technology Co., Ltd. TiO_2, acetophenone (98%), ortho-nitroacetophenone (95%), meta-nitroacetophenone (98%), ethyl-4-aminobenzoate (98%), 1-phenylethanol (97%) and benzyl alcohol (97%) were obtained from Acros. Meta-aminoacetophenone (97%) was offered by Accela. Hydroazobenzene (98%) and 4-nitrostyrene (95%) was purchased from TCI. All chemicals were used without further purification.

Double distilled water was used throughout the experiments.

Characterization of the catalysts

The transmission electron microscopy (TEM) images of the catalysts were obtained using a TEM JEOL-1011 with an accelerating voltage of 120 kV. The sample was dispersed in ethanol with the aid of sonication and dropped on an amorphous carbon film supported on a copper grid for the TEM analysis. The contents of metals in the catalysts were determined by ICP-AES method (VISTA-MPX).

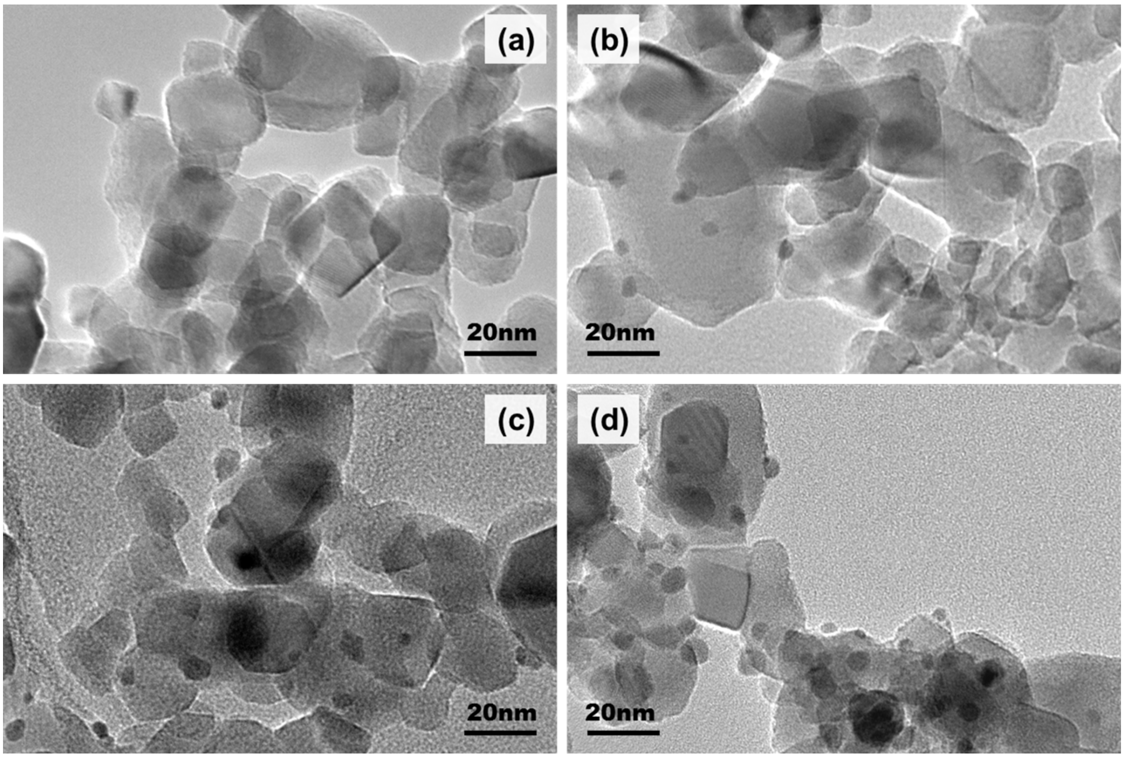

Fig. S1. TEM images of bare TiO_2 and TiO_2 with different Pd contents. (a)TiO_2; (b) Pd/TiO_2 with 1 wt% Pd; (c) Pd/TiO_2 with 2 wt% Pd; (d) Pd/TiO_2 with 3 wt% Pd.

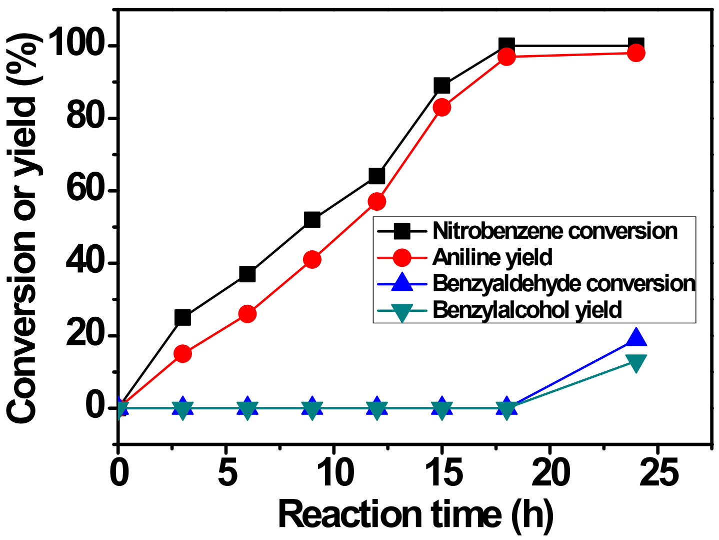

Fig. S2. Kinetic experiments for the photocatalytic hydrogenation of the mixture of nitrobenzene and benzyaldehyde. Reaction conditions: nitrobenzene, 0.1 mmol; benzyaldehyde, 0.1 mmol; glucose, 0.5 mmol; water, 1 mL; temperature, 25 ^{o}C; 0.025 g Pd/TiO_2 with 2wt% Pd; UV-light irradiation (350 nm, 4 mW/cm^2); illuminated area, 2 cm^2.

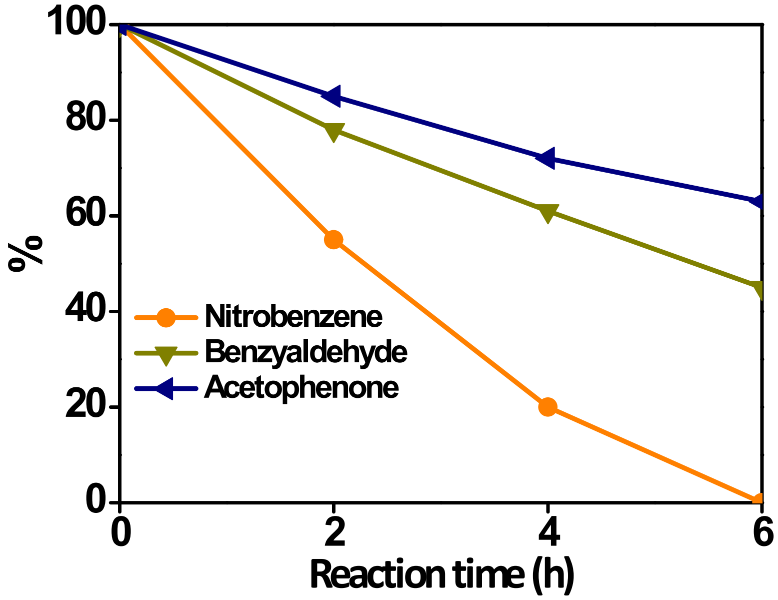

Fig. S3. Photocatalytic behavior of different substrates over Pd/TiO_2. Reaction conditions: substrates, 0.1 mmol; glucose, 10 mmol; water, 5 mL; temperature, 25 ^{o}C; 0.025 g Pd/TiO_2 with 2wt% Pd; UV-light irradiation (350 nm, 4 mW/cm^2); illuminated area, 2 cm^2.

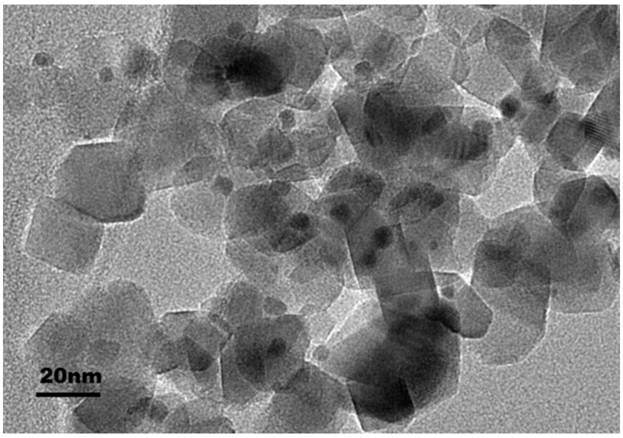

Fig. S4. TEM image of TiO_2 with 2 wt% Pd after four cycles reuse.
